# Person and Family Centeredness in Ethiopian Cancer Care: Proposal for a Project for Improving Communication, Ethics, Decision Making, and Health

**DOI:** 10.2196/16493

**Published:** 2020-05-19

**Authors:** Nataliya Berbyuk Lindström, Aynalem Abraha Woldemariam, Abebe Bekele, Christian Munthe, Rune Andersson, Bethlehem Girma Kebede, Barbro Linderholm, Wondemagegnhu Tigeneh

**Affiliations:** 1 Department of Applied Information Technology University of Gothenburg Gothenburg Sweden; 2 Department of Oncology and Radiotherapy School of Medicine, Health Science College Addis Ababa University Addis Ababa Ethiopia; 3 Department of Surgery, School of Medicine, Addis Ababa University Addis Ababa Ethiopia; 4 Department of Philosophy, Linguistics and Theory of Science University of Gothenburg Gothenburg Sweden; 5 Department of Infectious Diseases, Institute of Biomedicine Sahlgrenska Academy, University of Gothenburg Gothenburg Sweden; 6 Department of Oncology, Institute of Clinical Sciences University of Gothenburg and Sahlgrenska University Hospital Gothenburg Sweden

**Keywords:** communication, culture, cancer, ethics, person-centered care, Ethiopia

## Abstract

**Background:**

Cancer is a major burden in Ethiopia. The Oncology Department of Tikur Anbessa (Black Lion) Specialized Hospital in Addis Ababa is the sole specialist unit for cancer care in the country. With only a handful of oncologists, a lack of resources, and a huge patient load, the work is challenging, especially in terms of achieving effective and ethical patient consultations. Patients, usually accompanied by family members, often wait for a long time to receive medical attention and frequently depart without treatment. Handling consultations effectively is essential to help patients as much as possible within such limitations.

**Objective:**

The project has the following three main aims: (1) to enhance and expand the understanding of communicative and associated ethical challenges in Ethiopian cancer care; (2) to enhance and expand the understanding of the implications and use of person- and family-centered solutions to address such communicative challenges in practice; and (3) to plan and evaluate interventions in this area.

**Methods:**

This project develops and consolidates a research collaboration to better understand and mitigate the communicative challenges in Ethiopian cancer care, with a focus on the handling and sharing of decision making and ethical tension among patients, staff, and family. Using theoretical models from linguistics, health communication, and health care ethics, multiple sources of data will be analyzed. Data sources currently include semistructured interviews with Ethiopian staff (n= 16), patients (n= 54), and family caregivers (n= 22); survey data on cancer awareness (n=150) and attitudes toward breaking bad news (n=450); and video recordings of medical consultations (n=45). In addition, we will develop clinical and methodological solutions to formulate educational interventions.

**Results:**

The project was awarded funding by the Swedish Research Council in December 2017 for the period 2018 to 2021. The research ethics boards in Sweden and Ethiopia approved the project in May 2018. The results of the studies will be published in 2020 and 2021.

**Conclusions:**

The project is the first step toward providing unique and seminal knowledge for the specific context of Ethiopia in the areas of physician-patient communication research and ethics. It contributes to the understanding of the complexity of the role of family and ethical challenges in relation to patient involvement and decision making in Ethiopia. Improved knowledge in this area can provide a fundamental model for ways to improve cancer care in many other low-resource settings in Africa and the Middle East, which share central cultural prerequisites, such as a strong patriarchal family structure, along with strong and devout religiosity. The project will also serve to develop greater understanding about the current challenges in Western health systems associated with greater family and patient participation in decision making. In addition, the project will contribute to improving the education of Ethiopian health professionals working in cancer care by developing a training program to help them better understand and respond to identified challenges associated with communication.

**International Registered Report Identifier (IRRID):**

DERR1-10.2196/16493

## Introduction

### Background

The purpose of this project is to develop and consolidate a research collaboration between Sweden and Ethiopia to better understand and mitigate the communicative challenges in Ethiopian cancer care, with the aim of developing greater capacity and tools to address the identified problems. The project is a research collaboration between the School of Medicine and Tikur Anbessa (Black Lion) Specialized Hospital (TASH), Addis Ababa University in Ethiopia and the Department of Applied Information Technology, the Department of Philosophy, Linguistics and Theory of Science, and the Sahlgrenska Academy, University of Gothenburg, as well as the Sahlgrenska University Hospital in Sweden.

Through participating project investigators and researchers, as well as methodological and thematic connections and overlaps, the group will be associated with the International Network on Ethics of Families and the FORTE/VR research program “Addressing Ethical Obstacles to Person Centered Care” at Karolinska Institute, Stockholm, Sweden.

Cancer is killing more people in the developing world than HIV/AIDS, tuberculosis, and malaria combined [1-3], and it is a growing concern in Ethiopia [2,4]. Preventive actions, such as vaccination, or the prevention of cancer-inducing infections, such as human papillomavirus and HIV, and lifestyle adjustments to avoid cancer are generally rare [5]. The Oncology Department of TASH in Addis Ababa is the only facility that provides radiotherapy services to cancer patients in Ethiopia [6]. TASH currently treats about 10,000 patients per year; however, the estimated annual incidence of cancer is over 60,000 cases [7-9].

With few senior oncologists, only a couple of junior physicians, a lack of resources (at the hospital as well as among patients), and an enormous patient load from the entire country, the work environment is challenging, and it especially affects the prerequisites for effective and ethical consultations [10,11]. Patients wait for weeks to receive medical attention, and many depart without receiving any therapeutic or preventive assistance. Patients are usually accompanied by several family members, who are present when initial information is given and the results from diagnosis and treatment options are discussed. Handling consultations effectively is essential in order to help as many patients as possible within the mentioned limitations. It is also critical for many patients who cannot afford high-end treatments and who have to resort to simple self-care solutions at home in environments that are very different from the modern health care environments offered at TASH.

### Field Survey

Little is known about physician-patient communication in Ethiopia in general and in cancer care in particular [12,13]. In 2015, a minor field study about physician-patient communication in cancer care in Ethiopia was carried out [14]. The findings showed that although health care staff, patients, and caregivers were satisfied with their communication, more patient involvement in decision making was desired. Linguistic and cultural diversity, as well as the socioeconomic status of patients complicate physician-patient communication. Further, professionals and students report scant training on communication and learning skills, and even less training on how to manage the resulting ethical challenges.

Person or patient centeredness and shared decision making are important benchmarks for the quality of health care, and communication is vital for achieving patient-centered care (PCC) [15-17]. In cancer care, these aspects are paramount for health professionals to design care interventions and home care and for patients and family caregivers to understand prognoses, treatment options, and possible side effects during the stages of treatment (ie, testing, treatment, and follow-up) [18]. At the same time, the implementation of PCC has been proven to raise complex challenges, where communication problems and ethical issues come together in ways previously unseen, as the notion of PCC also includes the idea of increased patient participation in and power over care decisions [18-20]. Specifically, complex problems arise when patients with a minority immigrant background are undertaking or involved in home care or self-care [21,22], where standard communication strategies may become counterproductive. A further complication arises when the care situation is characterized by the strong involvement of family members [17,23-25], which is common in the Ethiopian setting [26-28] and markedly different from the typical Western care situation. In addition, strong religious norms increase the potential for conflicts concerning what is ethically acceptable in communicative processes among patients, family members, and health care staff.

At the same time, enhanced communication is essential for a successful physician-patient relationship, and patient involvement and shared decision making may have positive effects on health outcomes. If nothing else, these aspects are critical for the ability of the patient and family to approach the situation in a more informed way, thereby being better equipped to adapt and implement self-care [29].

The project’s inception was associated with a need that was identified by the staff at the TASH cancer clinic, and as a collective research team, we will explore ways to analyze the communication of staff with cancer patients and family caregivers, the handling and sharing of linked decision making, and the ethical tensions among patients, staff, and family. We focus on the influences of family structures, culture, gender, and socioeconomic status, as well as the dire economic and institutional situation of Ethiopian health care. Although the project is unable to influence changes in these important structural background factors, its aim is to help health professionals reduce the effects of the burden of poverty on Ethiopian cancer patients and their families.

Ethiopia is a multicultural society with more than 80 different ethnic groups, 83 languages, and over 200 different dialects [30]. Although the majority of patients visiting TASH report coming from Addis Ababa, patients from a number of regions in Ethiopia, such as Oromio, Amhara, Tigray, and the Southern Nations, Nationalities, and Peoples’ Region, are represented as well [1]. Language problems and cultural differences, reflected in views on health, illness, and treatment expectations [31,32], often complicate communication and necessitate the use of interpreters. When caregivers are interpreters, patients are less involved in consultations and decision making [14,33]. In addition, many patients come from rural areas, are poor, and either have low education or are noneducated, which further complicates their communication with physicians.

In this project, special attention will be paid to gender. About 73% of cancer patients in TASH are female [1]. Ethiopia has some of the lowest gender equality performance indicators in sub-Saharan Africa [34]. Women are often uneducated and do not participate in decision making because of gender relations [35]. As identified in our field study, in medical consultations, male caregivers often make decisions on behalf of female patients. In this project, we aim to raise awareness of diversity issues, develop approaches to encourage greater patient involvement (especially for women) in decision making, and create a more person- and family-centered approach.

### Objectives

The project addresses the following three main aims: (1) to enhance and expand the understanding of communicative and associated ethical challenges in Ethiopian cancer care; (2) to enhance and expand the understanding of the implications and use of person- and family-centered solutions to address such communicative challenges in practice; and (3) to plan and evaluate interventions in this area.

The project’s aims will also expand the base and context for understanding and addressing health communication- and ethics-related issues in person- and family-centered care in the contexts of Sweden and other developed countries, where health care for immigrants from Africa and other developing countries with a strong family culture is delivered.

## Methods

### Pilot Project (2015)

#### Description

The beginning of this project involved a pilot project conducted in Addis Ababa, Ethiopia, in spring 2015, as a part of the Master’s in Communication thesis of Kebede [14]. The study consisted of the following two parts: a qualitative study about communication in cancer care and a quantitative study about cancer awareness among the general public.

#### Qualitative Study About Communication in Cancer Care

A qualitative study was conducted at the Chemotherapy and Radiotherapy Center of TASH in Addis Ababa in 2015 by GK and BL. The primary focus was on how physicians, cancer patients, and family caregivers experience communication during consultations. Patient and family involvement in decision making, breaking of bad news, and related communication problems experienced in consultations were particularly emphasized.

The participants in the pilot study (physicians, patients, and family caregivers) were purposively sampled [36]. We aimed to attain a heterogeneous sample in terms of gender, cancer type (patients), and work experience (physicians).

In the pilot study, we adopted an ethnographic explorative qualitative study design, using triangulation of data collection that combined semistructured interviews with direct observations and video recordings of authentic interactions among physicians, patients, and family caregivers during hospital rounds. We aimed to obtain as complete a picture as possible of the challenges associated with communication in cancer care [37].

#### Quantitative Study About Public Awareness of Cancer in Ethiopia

In the pilot study, we also aimed to explore the level of public awareness of cancer in Ethiopia by undertaking a survey on 150 randomly chosen adult Ethiopians. Participants from the general public (aged above 18 years) were chosen using convenience sampling. The information from both studies was only partially analyzed, and it serves as an initial starting point for work in this project.

### Project Plan (2018-2021)

#### Description

The project is scheduled to occur over a 4-year period (2018-2021). The project's research team members are from the University of Gothenburg and the Sahlgrenska University Hospital in Sweden and from Addis Ababa University and TASH in Ethiopia. Each of the project investigators has allocated existing human resources in relevant disciplines to the project, including senior collaborators and junior assistants, as well as PhD and master’s-level students.

The three main aims of the project will be addressed in parallel and undertaken through a series of joint workshops. In between the joint workshops, the respective research teams will work on data analysis, methodology development, and development of interventions for improving physician-patient communication. The aim is to have 1 to 2 joint workshops per year across a 4-year period and to alternate these between Addis Ababa and Gothenburg. We prioritize the needs of Ethiopian professionals, which form the project's starting point, albeit also creating valuable input for the Swedish health care and research contexts. The project has been in progress for 2 years, with 2 years remaining for completion. Below, the project phases are presented.

#### Phase I: Project Commencement

##### Workshop 1 (Spring 2018)

The project commenced in Gothenburg. There were initial briefings on the Ethiopian situation and on current knowledge regarding health communication, person- and family-centered care, knowledge transfer in global health, and associated ethical challenges. The workshop involved initiation of the analysis of existing data, as well as planning of the work required and division of tasks. Additionally, it involved joint discussions of the methodology for future data collection and planning of research studies. The ethics application was submitted for the analysis of existing data.

The key project activities following Workshop 1 were as follows: (1) Deciding on the initiation of six research studies in the project (Table 1); (2) Conducting Study 1 involving analysis of the interviews and video recordings of hospital rounds from the pilot study. The audio-recorded interviews were transcribed verbatim and translated into English. Thematic content analysis [38] was used for data analysis. The video recordings were translated from Amharic into English and transcribed using a simplified version of the Gothenburg Transcription Standard [39]; and (3) Planning a communication training program for health care staff in Ethiopia in 2020.

**Table 1:** Overview of research studies.

Study #	Title	Data collection/analysis	Period
1	Explorative study of communication in cancer care (a general overview of the communicative challenges in cancer care in Ethiopia)	Analysis of the interviews and video recordings from the pilot project	2018-2019
2	Awareness of cancer in Ethiopia	Analysis of the survey data from the pilot project	2019-2020
3	Shared decision making in cancer consultations: A qualitative study	Conduction and analysis of video recordings of medical consultations	2019-2020
4	Attitudes toward shared decision making and breaking bad news: patients, family caregivers, and the general public	Survey to patients, family caregivers, and the general public	2019-2020
5	Attitudes toward shared decision making and breaking bad news: health care staff	Survey to health care staff	2020-2021
6	Development of a model of decision making in cancer care	Analytical model based on the results of the studies and discussions with research team members	2018-2021

The project was submitted for ethics approval to the Ethical Review Board of Western Sweden and the institutional review board (IRB) at the Department of Oncology, School of Medicine, College of Health Sciences, Addis Ababa University. Ethical challenges associated with data collection for the project included placing minimal burden on research participants and securing informed voluntary participation for patient consultations and interviews with staff, which were to be recorded. As the project includes ethical issues among its research questions, it will, of course, actualize these as part of the study and endeavor to contribute to a better understanding of ethical challenges in Ethiopian cancer care, as well as further inform about ethical theorizing for person-centered care in a Western immigrant context.

##### Workshop 2 (Spring 2019)

The workshop was conducted in Addis Ababa. There was a site visit to TASH and a discussion with the staff, as well as on-site researchers with an interest in health communication and related ethics. The workshop involved a presentation of the state of ongoing data analysis, discussion of how person- and family-centered care solutions could be applied in the Ethiopian context, and identification of possible hurdles. There was a continued discussion about data collection and intervention design. Additionally, it involved identification of possible adaptions of person-centered care solutions and development of methodological solutions in meetings with Ethiopian researchers, PhD students, and health care staff.

The key project activities following Workshop 2 were as follows: (1) Conducting Study 2 involving analysis of survey data on cancer awareness in Ethiopia. Simple descriptive statistics were used for the analysis; (2) Conducting Study 3 involving video recordings of medical consultations. Twenty-four physician-patient consultations were video recorded to study decision making and family involvement. It involved an initial analysis of data (transcriptions and quality check); (3) Conducting Study 4 involving the development of a survey about attitudes toward shared decision making and breaking bad news (patients, family caregivers, and the general public). It involved piloting and conducting the survey. The study used a comparative cross-sectional design to analyze the similarities and differences in attitudes toward the disclosure of clinical information to cancer patients and the inclusion of relatives in that process, among the following three groups: cancer patients, relatives, and the general public (150 participants per cohort, 450 in total). The data were coded, cleaned, edited, and entered into EpiData version 3.1 (EpiData Association, Odense, Denmark) to minimize logical errors. Thereafter, the data were exported to SPSS Windows Version 25 (IBM Corp, Armonk, New York, USA) for analysis. The analysis was undertaken by computing proportions and summary statistics for the three groups (cancer patients, relatives, and the general public). The attitudes of each group toward the disclosure of cancer status and family involvement were compared using the chi-square test; and (4) Initiating Study 6 involving the development of a model for shared decision making.

#### Phase II: Data Analysis and Securing Mutual Benefits

##### Workshop 3 (Spring 2020)

The workshop will be conducted in Gothenburg. Consultants and suitable specialists from relevant programs and networks in Scandinavian countries will be invited to attend this workshop to expand the Ethiopian partners’ professional networks. This workshop will concentrate entirely on the issues faced by the Ethiopian partners and will involve further discussion of the outcomes of data analysis from Phase I, methodological developments for data collection and analysis, and initial planning for the proposed publications.

The key project activities following Workshop 3 are as follows: (1) Submitting the manuscript of Study 1 to *PLOS ONE*; (2) Drafting the manuscript of Study 4 for submission; (3) Finalizing the analyses for Studies 2 and 3; (4) Conducting Study 5 involving the development of a survey for health care staff about attitudes toward shared decision making and breaking bad news, piloting and conducting the survey, and drafting a manuscript for submission to *Patient Education and Counselling*; (5) Continuing discussion of the model of decision making in cancer care (Study 6); and (6) Identifying possible adaptions of person-centered care solutions, developing methodological solutions for data collection and analysis, and planning communication training in Ethiopia.

##### Workshop 4 (Autumn 2020)

This workshop will be conducted in Addis Ababa. It will involve topical focus on how the Ethiopian experience can benefit health communication in the contexts of Sweden and other developed countries. Additionally, it will involve continued discussion of the data analysis and publication drafts. Communication training based on results from the conducted studies will be provided to medical students and health care staff in TASH. The developed model of decision making will be tested in the training course.

The key project activities following Workshop 4 are as follows: (1) Drafting manuscripts for papers related to Studies 2 and 3; (2) Preparing publication drafts related to Studies 5 and 6; and (3) Further developing the communication training course.

#### Phase III: Project Completion

##### Workshop 5 (Spring 2021)

This workshop will be conducted in Addis Ababa. It will involve finalizing publications from the current project, as well as discussion of a new project plan for future data collection and interventions. Additionally, it will involve briefing and discussion with local health care staff and networks about the current project’s results, as well as development of a new project plan for future data collection and interventions.

The key project activities following Workshop 5 are as follows: (1) Submitting publications to journals in the areas of health care communication, public health, and oncology and (2) Revising a new project plan for future expanded data collection and intervention studies.

##### Workshop 6 (Autumn 2021)

The concluding workshop will be conducted in Gothenburg. It will involve continued briefing and discussion with local health care staff and networks about the current project’s results, as well as submission of a new project application.

The key project activity following Workshop 6 is as follows: Finalizing and submitting a new project application for expanded data collection and intervention studies.

## Results

This study was awarded funding by the Swedish Research Council in December 2017 for the period 2018 to 2021. Ethics approval was obtained from the Ethical Review Board of TASH for the pilot project (April 14, 2015). The research ethics boards in Sweden and Ethiopia approved the project in May 2018 (DNR 520-18 and ONC IRB 27, respectively). The results of the studies will be published in 2020 and 2021.

## Discussion

This study contributes to the understanding of the complexity of the role of family, along with patients’ dependency on family members for communication, support, and access to care, which creates particular ethical dilemmas for medical staff. Better understanding of communication and the factors that influence cancer patients’ health-seeking behaviors and adherence to treatment can lead to improved health care services and better handling of health care ethical challenges in this context.

This project is the first step toward providing unique and seminal knowledge for the specific context of Ethiopia in the areas of physician-patient communication research and ethics. The project also contributes to the quality of cancer care in developing country settings and does so by deepening the understanding of key practical and theoretical challenges in physician-patient communication through sharing of expertise from Sweden to Ethiopia in ways that are designed to leave a lasting impact. The Ethiopian case can be a foundational model for improving communication in cancer care in other low-resource settings, which share central cultural prerequisites, such as a strong patriarchal family structure, along with strong and devout religiosity.

The project also aims to improve the education of health professionals and medical students in Ethiopia by developing a communication training course, with a focus on ethical aspects and shared decision making. We believe that this work will have important contributions to mainstream research in health communication, health care ethics, and global health.

Finally, communication and interaction among health professionals, patients, and family caregivers actualize a broad spectrum of welfare considerations particularly regarding how to prioritize between different needs within health care and within families and how to handle the ensuing ethical dilemmas that each of these groups face. The project will also serve to develop further understanding about current challenges in Western health systems associated with greater family and patient participation in decision making.

## Appendix

Multimedia Appendix 1 Previous peer-review reports: Final statement from the review panel of the Swedish Research Council (Review panel: UF-5).

## Abbreviations

IRB: institutional review board
PCC: patient-centered care
TASH: Tikur Anbessa (Black Lion) Specialized Hospital
