# Supplementary material for: Person and Family Centeredness in Ethiopian Cancer Care: Proposal for a Project for Improving Communication, Ethics, Decision Making, and Health
Source: JMIR Res Protoc. 2020 May 19;9(5):e16493. doi: 10.2196/16493 (PMC7267985; doi:10.2196/16493)
Supplement: Multimedia Appendix 1 [file resprot_v9i5e16493_app1.pdf]

Vetenskapsrådet

Final statement from review panel

**2017-05410**	**Nataliya Berbyuk Lindström**	**Review panel: UF-5**

Call name: Research Grants Open call 2017 (Development Research)

Project title (english): Person- and Family-Centeredness in Ethiopian Cancer Care: Improving Communication, Ethics, Decision-making and Health

Type of grant: Research Network Grant

Focus: Swedish Research Links

Novelty and originality **6**

1 - Poor, 2 - Weak, 3 - Good, 4 - Very Good, 5 - Very good to excellent, 6 - Excellent, 7 - Outstanding

This an application much well addressing a new WHO-priority focusing on patient centered care and quality of care in low-income countries. The research collaboration predicts a most valuable research experience that can generate seminal knowledge of general importance for low-income countries as well as south-to-north transfer of knowledge in a global world.

Scientific quality of the proposed research **5**

1 - Poor, 2 - Weak, 3 - Good, 4 - Very Good, 5 - Very good to excellent, 6 - Excellent, 7 - Outstanding

This project "Person- and Family-Centeredness in Ethiopian Cancer Care: Improving Communication, Ethics, Decisionmaking and Health" departs from an already conducted data collection and established research collaboration between health care departments and universities in Ethiopia and Sweden.
The application will address how to enhance and expand the understanding of communicative and associated ethical challenges in Ethiopian cancer care. Methodology to address this objective are most appripately described: joint analysis and processing of already collected data, methodological development and workshops and eventually collection of new data. The project ultimately aims to develop clinical and methodological solutions to prepare more expanded data collection and interventions in this area.

Merits of the applicant(s) **6**

1 - Poor, 2 - Weak, 3 - Good, 4 - Very Good, 5 - Very good to excellent, 6 - Excellent, 7 - Outstanding

This a research team most qualified to pursue the research colaboration representing competences in linguistic, health communication, ethics, global health and clinical medicine. International and national research collaboration within the research area is further foreseen.

Feasibility **3**

1 - Not feasible, 2 - Partly feasible, 3 - Feasible

The research application is in detail describing the steps to address the resarch aims. The infrastructure and the network are well described, part of the data is already collected and will suffice to fullfill the objective.

Complementarity of the research (the added value of the research collaboration) **6**

1 - Poor, 2 - Weak, 3 - Good, 4 - Very good, 5 - Very good to excellent, 6 - Excellent, 7 - Outstanding

The selection of team members are most appropriate and multidisciplinary. The collaboration will lead to transfer of knowledge and have mutual benefits, both north-to-south and south-to-north.

Overall assessment of the application's scientific quality* **6**

1 - Poor, 2 - Weak, 3 - Good, 4 - Very good, 5 - Very good to excellent, 6 - Excellent, 7 - Outstanding

Within the research area this is a high quality project, outlining a research process departing form already collected data that will have several challenges that the applicant are well describing, aiming to serve as founding for further tudies and inveterventional studies and changes in care practices. The multi-disciplinary research process is excellently described.

Relevance for call* **3**

1 - Not relevant, 2 - Relevant, 3 - Very relevant

According to the new WHO priorities this project is front-line resarch addressing patient-centered-care and quality of care.
